# Mediating role of perceived social support in the relationship between unemployment and mental distress among healthcare graduates during the COVID-19 era

**DOI:** 10.3389/fpubh.2024.1490004

**Published:** 2025-01-28

**Authors:** Lea John, María Teresa Solís-Soto, Katja Radon

**Affiliations:** ^1^Institute and Clinic for Occupational, Social and Environmental Medicine, University Hospital of LMU Munich, Munich, Germany; ^2^Center for International Health^LMU^ at Institute and Clinic for Occupational, Social and Environmental Medicine, University Hospital of LMU Munich, Munich, Germany; ^3^OH TARGET Competence Center, Universidad San Francisco Xavier de Chuquisaca, Sucre, Bolivia

**Keywords:** perceived social support, unemployment, mental distress, healthcare graduates, COVID-19, mediation analysis

## Abstract

**Objective:**

This study investigates the mediating role of perceived social support in the relationship between unemployment and mental distress among young healthcare graduates in Bolivia during the COVID-19 pandemic.

**Methods:**

A cross-sectional analysis within a cohort study was conducted using data from 109 healthcare graduates from Bolivia collected through an online survey in 2022. The survey measured employment status, mental distress with the 12-item General Health Questionnaire (GHQ-12), and perceived social support using the Multidimensional Scale of Perceived Social Support (MSPSS). Mediation analysis was performed in R to examine the mediating effect of perceived social support on the relationship between unemployment and mental distress.

**Results:**

More than two-thirds of participants reported mental distress. Consistent with the main effect model, employment was directly associated with lower levels of mental distress, and perceived social support was positively related to better mental health. However, perceived social support did not statistically significant mediate the impact of unemployment on mental distress, with only 2.1% of the effect being mediated through perceived social support.

**Conclusion:**

Despite the beneficial effect of perceived social support on mental health, it did not significantly mediate the relationship between unemployment and mental distress among Bolivian healthcare graduates during COVID-19. The findings highlight the need for targeted mental health support that go beyond social support for unemployed healthcare graduates during crises.

## 1 Introduction

In March 2020, the emergence of COVID-19 marked the beginning of a major public health event that precipitated widespread economic, social, political, and health crises (1, 2). The pandemic’s multifaceted impact has contributed significantly to the development of mental distress within the population (3). Mental distress is recognized as a public health concern, as it not only reduces the quality of life but also impacts health outcomes including a reduction in quality of life and increased mortality rates (4).

The COVID-19 pandemic has severely impacted healthcare workers, particularly younger individuals still in training or recently graduated, making them more vulnerable to mental distress (2, 5). For healthcare workers, factors such as the collapse of the healthcare system, extended working hours, continuous exposure to the virus, lack of personal protective equipment, and instances of disrespect and violence contributed to the decline in mental health during the pandemic (2, 6).

Healthcare workers across the globe reported increases in post-traumatic stress disorder, anxiety, depression, psychological distress (7, 8) job burnout (8), fatigue, and loneliness (6). In Latin America, a large number of studies have examined these issues, consistently identifying higher levels of anxiety, depression, stress, and mental distress among healthcare workers (2, 9). Prevalence estimates, however, vary widely, reflecting differences in study methodologies and survey instruments. In Bolivia, findings from the COVID-19 Healthcare Workers Study (HEROES) revealed high levels of mental distress among healthcare workers (10). Unfortunately, only a minority of studies monitored changes of mental health using longitudinal designs (6, 11–15).

In Bolivia, secure employment opportunities are often limited, with informal employment constituting approximately 90% of all jobs (16). This employment insecurity, particularly acute during the COVID-19 pandemic (17), has intensified the experience of unemployment and economic instability (2). During the pandemic young healthcare graduates in Bolivia faced an employment crisis, with unemployment rates of 7.9% for the general population and 15.4% for those under 25 years in 2020 (17). Research indicates that people have worse mental health when they are unemployed compared to when they are employed (18), a trend that has been especially pronounced during the COVID-19 pandemic (19–22). Furthermore, the precariousness of employment, characterized by unstable, insecure, and poorly paid job conditions, has been exacerbated by COVID-19, particularly for those employed in the healthcare sector (23).

Perceived social support has consistently been highlighted as a protective factor to mental health-related outcomes (24). Perceived social support is defined as an individual’s expectation and assessment of available social networks when needed (20). In stressful circumstances and situations perceived social support can affect mental health through direct or indirect pathways (25). Examples for such circumstances and situations are precarious employment (24), job burnout (26), job insecurity (27), unemployment (22) and the COVID-19 pandemic (28). The direct pathway, also called the main effect model, posits that perceived social support has a direct impact on individual’s mental health (29). According to the indirect pathway, also called the buffering effect model, perceived social support can buffer the negative impact of stressful situations, e.g., unemployment, on the mental state of individuals (26). Evidence from meta-analytic studies suggests that the main effects of perceived social support on mental health are more consistently observed than buffering (indirect) effects (30, 31).

Despite extensive research on the role of perceived social support in mental health, there remain critical gaps, particularly concerning unemployment among young healthcare graduates from lower-middle-income countries (LMIC) such as Bolivia during the COVID-19 pandemic. The pandemic introduced new dynamics to the relationship between perceived social support and mental health, which have been investigated mainly in high-income countries (HIC) and in limited contexts. Individual social networks depend on the social and cultural context where individuals live and grow up (32). In Latin America, where economic security, social protection, and services are limited, the strong interdependence within social networks makes perceived social support particularly essential (32, 33). There is evidence, that the support of family members and friends play an important role in the transition from academic to employment life (28). However, we hypothesize that the strain on social support networks during the COVID-19 pandemic have altered the impact of unemployment through perceived social support on mental distress. The described cultural and socioeconomic context makes Bolivia a particularly relevant setting for examining how perceived social support interacts with unemployment and mental distress during crises such as the COVID-19 pandemic.

The aim of this study is to explore the mediating role of perceived social support in the relationship between unemployment and mental distress among healthcare graduates in Bolivia during the COVID-19 pandemic. The findings are of public health relevance, providing crucial insights for developing targeted interventions that address mental health challenges during crises. Understanding these dynamics can inform public health strategies to strengthen support mechanisms and mitigate the mental health effects of unemployment in LMIC like Bolivia.

## 2 Materials and methods

### 2.1 Study design and participants

A cross-sectional analysis within a cohort study (34) was conducted in 2022. Data was collected through a pseudo-anonymous online survey where personal information (e.g., names) was collected but linked to the survey responses only via a unique identifier. The personal data and responses were stored separately, ensuring that participants’ identities could not be directly traced without the identifier. Participants were informed that they could withdraw from the study at any point without providing a reason.

**Figure 1:**
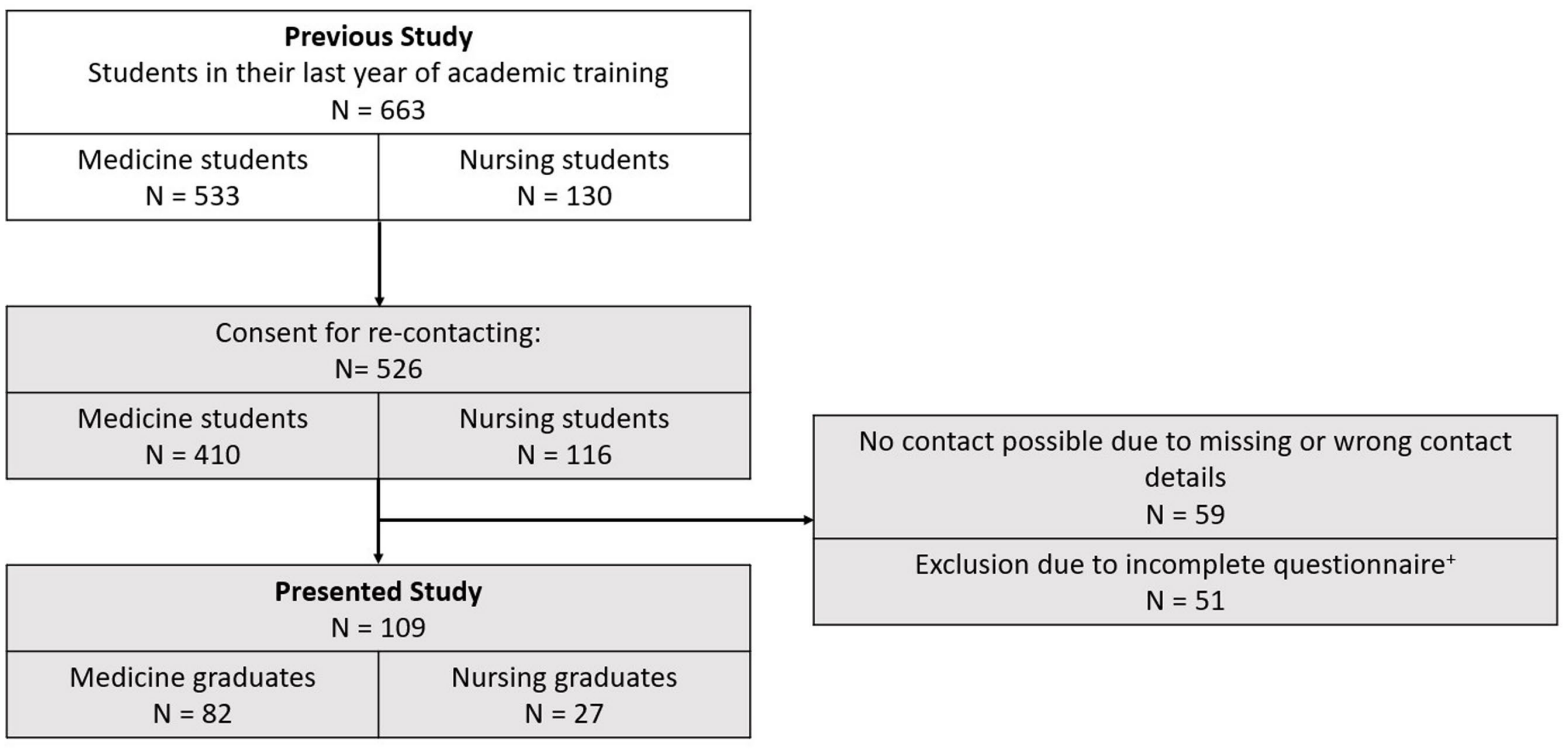
Number of participants in previous and current studies. ^+^Exclusion criteria: participants were excluded if any of the following variables were incomplete: employment status, perceived social support, or mental distress.

Participants were graduates from the Universidad San Francisco Xavier de Chuquisaca (USFX) in Sucre, Bolivia. Participants were recruited in 2018, when they were in their final year of academic training in medicine or nursing. A total of 526 individuals from this previous study, who had agreed to be re-contacted, were invited to participate in this study. Of those who could be reached, 109 participants (23.3%) completed the questionnaire (Figure 1). Of the final sample, 27 participants (24.8%) were men, and 82 participants (75.2%) were women. The average age of participants in 2022 was 27.3 years (standard deviation (SD) = 2.9). All participants provided written informed consent before taking part. A reminder was sent after one, three, and four weeks of initial sending to ensure higher response. To incentivize participation, five Farmacorp vouchers worth 200 Bolivianos (approx. 29 USD) each were offered as prizes in a lottery draw.

Ethical approval for this study was obtained from the Ethical Committee of the Medical Faculty at the Universidad Mayor de San Simón on November 8, 2021, and from the Ethical Committee of the Medical Faculty at Ludwig Maximilian University in Munich on June 11, 2022 (project number 22–0451).

### 2.2 Questionnaire instruments and variable definition

The questionnaire was provided in Spanish via SurveyMonkey (Momentive Europe UC, Dublin, Ireland) and comprised the following components: socio-demographic characteristics, mental distress, employment status, precarious working conditions and perceived social support. All questions and scales included in the questionnaire were sourced from established and validated measurement instruments to ensure reliability and validity of the data collected.

The socio-demographic characteristics included the respondents’ gender (male, female, or diverse), age (continuous), and economic situation (good, neither good nor bad, or bad).

The employment status of respondents was assessed through the question, “Are you currently employed?” Those who responded in the negative were classified as unemployed and were invited to indicate the reasons for their current status. The following response options were provided: “I am looking for a job because I was unable to find one,” or “I am currently engaged in studies or training.” Those who responded in the affirmative were classified as employed and subsequently completed the Employment Precariousness Scale (EPRES) (35). The EPRES consists of the subscales temporariness (in terms of contract duration), disempowerment (in terms of employment conditions such as working hours and pay), vulnerability (in terms of treatment at work), wages (in terms of cost coverage), rights (in terms of benefits such as pensions), and exercise rights (in terms of holidays and sick leave), which sum up to an overall score between 1 and 4. Following a validation study from Chile (35), a (very) high level of precariousness (LoP) was defined as an overall mean ≥ 2.0. Due to the low sample size in the category of none LoP, the moderate and none LoP were combined and defined as an overall mean < 2.0.

Mental distress, the outcome variable, was assessed using the validated 12-item General Health Questionnaire (GHQ-12). For mental distress as continuous variable each item was scored on a scale from 0–3, with positive items coded as 0-1-2-3 and negative items as 3-2-1-0, resulting in a total score ranging from 0 to 36. For the description of the participants, bivariate analysis and for sensitivity analysis, a dichotomous scale (also known as binary scale) of GHQ-12 was applied for mental distress as binary variable, with positive items coded as 0-0-1-1 and negative items as 1-1-0-0, resulting in a score range of 0–12. Since there is no validation study available for Bolivia, a threshold score was adopted from a Chilean validation study (36). Participants with a GHQ-12 score greater than 4 (with mental distress as binary variable) were classified as having clinically relevant mental distress (subsequently referred to as ‘mental distress’). Sensitivity analysis were conducted with a threshold of 5/6.

Perceived social support, the mediating factor, was measured using the validated Multidimensional Scale of Perceived Social Support (MSPSS). This scale assesses the social support an individual perceives from three different sources: family, friends, and a significant other. The MSPSS consists of 12 items ranging from 1 (“very strongly disagree”) to 7 (“very strongly agree”). A higher overall score indicates a higher level of perceived social support. An overall score was used for the mediation analysis, as the subscales (family, friends, and significant others) are highly correlated and measure similar aspects of social support. However, for transparency we also report the subscales.

Gender and economic situation were treated as potential confounders in the analysis, given their likely association with both employment status and mental distress, as supported by evidence (34). Age was not considered a confounder due to the limited age range of participants.

### 2.3 Statistical analysis

Calculations were done in R (Version 4.1.1) (37). Descriptive statistics were applied to nominal and ordinal variables using absolute and relative frequencies, while metric variables were described as means with standard deviations. Group differences were assessed using Chi-Square tests for categorical variables, Fisher’s Exact Tests for small sample sizes, and t-tests for continuous variables. The Pearson’s correlation was utilized to assess relationships between the main variables. Mediation analysis was performed using the package MEDIATION (38) to explore the mediating effect of perceived social support (mediator) on the relationship between employment status (independent variable) and mental distress (dependent variable), with gender and economic situation as potential confounders. Bootstrapping was used due to its suitability for smaller sample sizes, ensuring robust estimation of effects. Sensitivity analysis were conducted to ensure the robustness of the findings. Multiple imputation (n = 20) was performed using the package MICE (39).

## 3 Results

### 3.1 Description of participants

Table 1 presents a description of the characteristics of the participants. A greater proportion of women and medicine graduates completed the questionnaire. The majority of participants described their economic situation as neither good nor bad. A smaller proportion indicated that their economic situation was good, while the fewest described it as bad. About half of the participants indicated that they were employed. Mental distress (as binary variable) was reported by more than two-thirds of the participants. When using a GHQ-12 score threshold of greater than 5 instead of 4, the analysis revealed a prevalence of 56.4% for mental distress among participants.

**Table 1:** Characteristics of the participants (*N* = 109).

Characteristics	Category	Missings	*N* (%)
Gender°	Men	0	27 (24.8)
	Women		82 (75.2)
Career	Nursing	0	27 (24.8)
	Medicine		82 (75.2)
Economic situation	Good	3	20 (18.9)
	Neither good, nor bad		70 (66.0)
	Bad		16 (15.1)
Employment	Yes	5	56 (53.8)
	No		48 (46.2)
Mental distress as binary variable^§^	Yes	8	69 (68.3)
	No		32 (31.7)
			Mean (SD)
Mental distress as continuous variable^#^		8	19.1 (2.2)
Age^+^		0	27.3 (2.9)

°No respondent indicated to have the gender “diverse”; ^§^Dichotomous scale for GHQ-12 with 0-0-1-1 for positive items and 1-1-0-0 for negative items, threshold > 4; ^#^Likert scale for GHQ-12 with 0-1-2-3 for positive items and 3-2-1-0 for negative items, ranging from 0 to 36; ^+^Age ranged from 23 to 37.

### 3.2 Description of mental distress

Participants with mental distress (68.3%) did not differ much from participants without mental distress regarding their gender, career, economic situation, employment status and age (Supplementary Table S1).

### 3.3 Description of employment status

More than half of the graduates were employed. Among the unemployed participants, 31.3% were in training, while 68.7% were actively seeking employment. Among those employed, nearly half (44.9%) reported a (very) high level of precariousness at work. In particular, conditions related to disempowerment and exercise rights had a negative impact on the precariousness at work. A detailed table can be found in the supplementary material (Supplementary Table S2).

### 3.4 Description of perceived social support

As shown in Table 2, participants generally perceived their social support as slightly positive, with average scores ranging between 4 (“neither agree nor disagree”) and 5 (“agree”). Support from family was rated the highest, followed by support from significant other. Support from friends was rated the lowest, although it remained within the positive range. A detailed table is provided in the supplementary material (Supplementary Table S3).

**Table 2:** Subscales of perceived social support measured with MSPSS of all participants (*N* = 109).

Subscales	Missings	Mean (SD)
Significant other	19	4.66 (1.47)
Family	18	4.73 (1.55)
Friends	18	4.30 (1.40)
Total	19	4.56 (1.23)

MSPSS = Multidimensional Perceived Social Support Scale; Scores: 1 “very strongly disagree,” 2 “strongly disagree,” 3 “disagree,” 4 “neither agree nor disagree,” 5 “agree,” 6 “strongly agree,” 7 “very strongly agree”; SD = standard deviation; for more details see Supplementary Table S3.

### 3.5 Correlation among study variables

Table 3 displays the correlations among the main variables employment status, perceived social support and mental distress (continuous variable). The results indicated that employment was not correlated with perceived social support. There was a negative association between employment and mental distress, indicating that employment was statistically significant linked with lower level of mental distress. Perceived social support was negatively statistically significant associated with mental distress meaning higher scores in perceived social support led to lower scores in mental distress.

**Table 3:** Correlations among main variables (*N* = 89°).

Variables	Employment	Perceived social support
Employment	1	
Perceived social support	−0.01	1
Mental distress^#^	−0.22*	−0.36*

^#^Mental distress as continuous variable; *p < 0.05; *N* = 89° as complete data analysis without imputation was conducted; Pearson’s correlation as variables are normally distributed.

**Figure 2:**
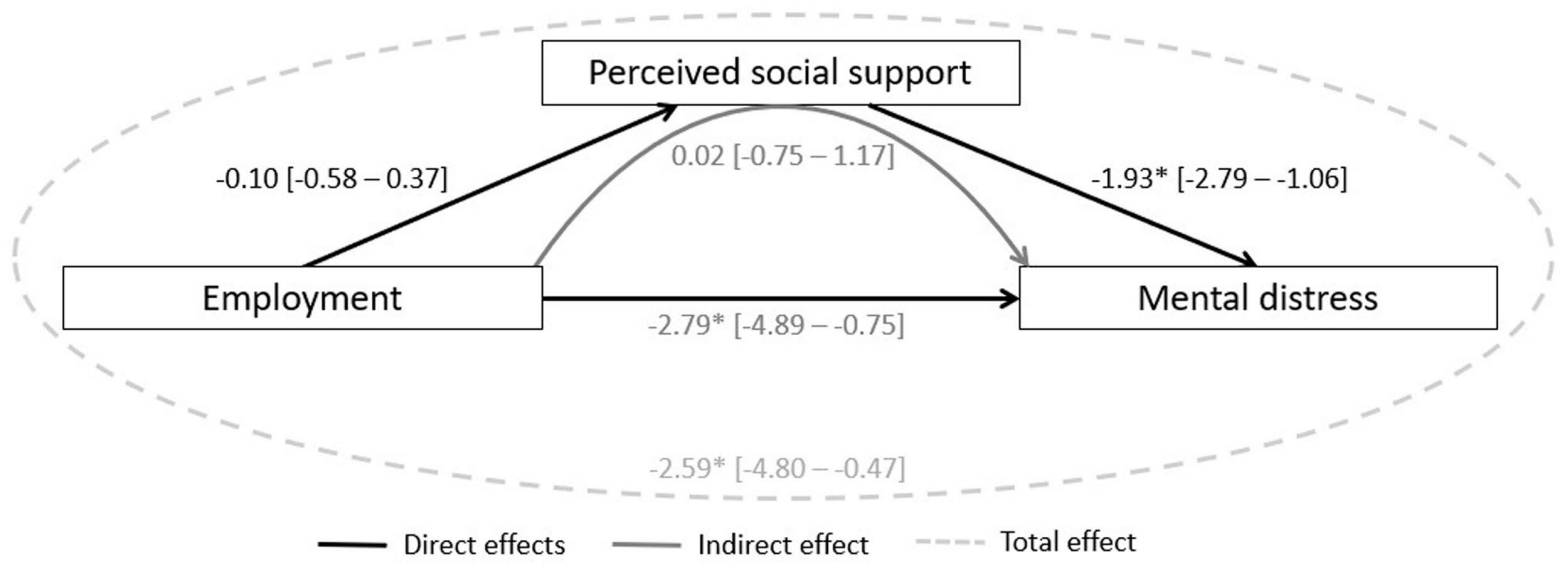
Mediating model of perceived social support between employment and mental distress (*N* = 109). Controlled for gender and economic situation; *p-value < 0.05; mental distress: continuous variable; imputed data; 95% confidence interval in brackets. Direct effects: measure the direct impact of employment status on mental distress, excluding any mediation by perceived social support. Measure the direct impact of employment status on perceived social support, as well as the direct impact of perceived social support on mental distress. Indirect effect: measures the impact of employment status on mental distress through the mediator, perceived social support. Total effect: Captures the overall relationship between employment and mental distress, including both direct and indirect effects.

As shown in Figure 2, the largest impact of employment on mental distress occurred directly, with a statistically significant effect that resulted in lower scores in mental distress, indicating a positive influence on mental distress. While perceived social support had a statistically significant effect on mental distress (direct effect), it did not statistically significantly mediate the effect of employment on mental distress (indirect effect). Overall, employment was associated with a reduction in mental distress scores, including both direct and indirect effects (total effect). About 2.1% of the total effect of employment on mental distress is mediated through perceived social support.

The sensitivity analysis indicated effects in the same direction with consistent significance levels (Supplementary Figures S1–S3). As shown in Supplementary Figure S1, we performed a complete case analysis without imputation, which resulted in consistent results. In Supplementary Figure S2, we used mental distress as binary variable with a threshold of 4/5, instead of mental distress as continuous variable, which yielded similar results. Additionally, we differentiated between two groups within the unemployed: those undergoing training and those actively seeking work. The results for those actively seeking work were consistent with Table 3, while no effects were observed for those in training (Supplementary Figure S3).

## 4 Discussion

This study is the first to explore the mediating role of perceived social support in the relationship between unemployment and mental distress among healthcare graduates in Bolivia during the COVID-19 pandemic. Our findings indicate that the prevalence of mental distress was high among participants, with unemployment being a statistically significant risk factor for an increase in mental distress. Our results suggest that, despite the general positive impact of perceived social support on mental distress, it does not mediate the impact of employment on mental health outcomes among healthcare graduates in Bolivia during COVID-19.

### 4.1 Consistency with other studies

#### 4.1.1 Mental distress

As highlighted in the article by John, L. et al. (34) on this cohort, our study found a high prevalence of mental distress among healthcare graduates, which was higher than in Bolivian teachers (43%) but lower than in Bolivian miners (81%) (40, 41). The elevated mental distress among miners is likely due to extreme working conditions such as extreme temperatures, noise, and shift work (41). In the study of Bolivian teachers, a threshold of 5/6 was used for mental distress. When applying the same threshold in our study, we still observed a higher prevalence of mental distress among healthcare graduates compared to teachers (40). This higher prevalence may be attributed to the sector-specific work environment and the impact of COVID-19, both of which exacerbated job demands and job insecurity (5). These factors are known to contribute to elevated levels of mental distress (19, 22). This is also reflected in the high ranking of precarious work as a contributing factor in our study.

Comparing our results to the HEROES study, which assessed mental health across 26 countries, including Bolivia, we found higher average GHQ-12 scores, with an average of 19.1 in our study versus 13.5–14.9 in the HEROES study for Bolivia (10). This difference may be explained by the older age of HEROES participants, as younger healthcare workers are generally considered to be more prone to mental distress (2). Additionally, the HEROES study included only employed participants, whereas our study included both employed and unemployed individuals, which likely contributed to the higher distress levels observed.

#### 4.1.2 Perceived social support

The average scores for perceived social support in this study (4 to 5 out of 7) indicate that participants view their perceived social support positively, though not exceptionally high. Our results align with findings from other Latin American studies (33, 42) and thus culturally similar populations. For example, a study from Colombia (42) highlights the family as a crucial source of support for young adults. Similarly, our study showed that the family is the primary source of support for healthcare graduates, followed by significant other. Similar results were found in research on Venezuelan migrants in Peru. The highest perceived social support came from family (5.71 (1.34)), followed by significant other (5.62 (1.44)) and friends (5.19 (1.44)) (33). The overall higher values compared to our study may be attributed to closer-knit social network structures in the migrant population, and the heightened reliance on social support during migration challenges (43). Additionally, our study, conducted during the COVID-19 pandemic, reflects the consistent importance of family support across Latin America while also highlighting the potential impact of the pandemic on perceived social support (28).

#### 4.1.3 Perceived social support and mental distress

Overall, our findings align with evidence, that perceived social support has a positive effect on mental distress (18, 24, 32). A Brazilian study emphasized the crucial role of support from extended family, children, and partners in reducing mental distress, while support from friends was found to be less significant (32).

#### 4.1.4 Employment status

We observed a high unemployment rate in our study compared to official numbers from Bolivia (17). The high unemployment rate observed among healthcare graduates could be influenced by sector-specific challenges not reflected in national statistics. Additionally, this discrepancy might be due to participants not disclosing informal employment, which comprises a substantial portion of the labor market in Bolivia (16). Many participants were also still in training. Sensitivity analysis indicate that excluding those still in training did not affect the overall results in the mediation model (Supplementary Figure S3).

#### 4.1.5 Employment status and perceived social support

We could not find a correlation between employment and perceived social support. Nevertheless, previous studies have suggested that perceived social support has the potential to influence the behavior of youth, which in turn can affect their work domain and employment status (44, 45). These discrepancies could stem from several factors, including cultural differences and the context of the COVID-19 pandemic. For example, in some cultures, social networks play a crucial role in providing support and shaping employment opportunities, while in other cultures, individuals may rely more on formal social services (46). A study on Mexican graduates found that 73% of participants secured employment after graduation due to their social networks (47). Additionally, the unique challenges and disruptions caused by the COVID-19 pandemic may have altered the dynamics between perceived social support and employment, leading to different outcomes compared to pre-COVID-19 studies.

#### 4.1.6 Employment status and mental distress

In Bolivia, the lack of a social welfare system means being unemployed is a threat for survival. This context amplifies the impact of employment on mental distress, compared to HIC where social safety nets offer more support (48–51). Our findings align with the well-researched negative effects of unemployment on mental distress (18, 19, 21, 52). For example, Medina Fernández, I.A. et al. examined the mental health of Mexican graduate students during the COVID-19 pandemic and found that those employed as healthcare workers experienced lower levels of mental distress compared to those who were unemployed (52). Furthermore, our sensitivity analysis revealed that no such effect was observed for participants who were still undergoing training, indicating that being in training does not contribute to increased mental distress (Supplementary Figure S3).

#### 4.1.7 Employment status, perceived social support and mental distress

In our study, we confirm the main effect model (25), which is predominantly supported in HIC (26, 30, 31), but not the buffering model. Thus, in the context of the COVID-19 pandemic, perceived social support does not appear to mediate the impact of employment on mental distress among young healthcare graduates in Bolivia. Most studies that found a mediating effect of perceived social support were conducted before the COVID-19 pandemic and in contexts outside of Latin America, which may explain the differing results due to cultural differences (26).

### 4.2 Limitations and strengths

The response rate in our study was comparably low, which means our results cannot be considered fully representative for the target population. This may partly be due to the unique challenges of conducting research during the COVID-19 pandemic and in LMICs like Bolivia. This limitation introduces the potential for selection bias, and the low statistical power may have contributed to the non-detection of relationships, such as the buffering effect, between the study variables. However, a non-responder analysis on this cohort conducted by John, L. et al. (34) found no statistically significant differences in gender distribution (women: 66.5% in the original sample, 75.9% in present sample) or age (original sample: 23.5 years, SD = 0.3; present sample in 2018: 24.0 years, SD = 0.1), among other key factors, suggesting that attrition did not systematically affect these factors.

Misclassification of exposure, mediator, and outcome may have occurred because these were assessed solely through online questionnaires. The GHQ-12, while a validated screening tool with a strong correlation to mental illness and predictive of future psychiatric diagnoses (53), is not a substitute for a clinical diagnosis (3). The MSPSS, also validated in Latin America (42), is limited to assessing emotional support and does not explicitly account for physical proximity as a source of support. This limitation restricted our ability to examine forms of support that rely on physical proximity, which is particularly relevant during a pandemic when physical distancing measures are in place (2). Additionally, we did not differentiate between formal and informal employment, which may have led to misclassification regarding the employment status. Also, we did not distinguish between frontline healthcare workers, which are workers providing direct care for COVID-19 patients, and non-frontline healthcare workers, although there is indication that the prevalence may be higher in frontline workers (9). Consequently, no dose–response relationship could be evaluated.

We acknowledge that not all of Baron and Kenny’s (54) criteria for mediation were fully met in our study. However, the use of bootstrapping offers a robust alternative, as it is particularly suited for detecting effects in smaller samples and under conditions where conventional assumptions may not hold (38).

A bidirectional relationship may arise, as individuals who are unemployed could already have poorer mental health and perceive less social support compared to those who are employed, which complicates causal inference (7). Moreover, since the study cohort consists of young people who typically perceive higher levels of social support (42), the results may not be generalizable to the broader population.

However, overall consistent results were observed in sensitivity analysis (Supplementary Figures S1–S3), reinforcing the reliability of our findings. Potential confounding by gender and economic situation were considered, although we missed other relevant factors like the duration of employment or unemployment and baseline mental health status. The economic situation in our study was assessed using a simple categorical scale (good, neither good nor bad, bad) rather than a continuous scale. While a continuous scale might have provided a more detailed picture of participants’ economic status, the categorical approach was chosen for its clarity and ease of interpretation. This categorization may limit the ability to fully capture the complexity of participants’ economic circumstances.

### 4.3 Implications for public health

The high prevalence of clinically relevant mental distress in this population underscores the urgent need for targeted public health interventions. Addressing unemployment as a significant risk factor requires creating stable employment opportunities and improving working conditions in the healthcare sector. Expanding access to professional mental health care, such as integrated mental health services in primary care and telemedicine platforms, can improve accessibility, especially in resource-limited LMICs. Strengthening broader structural support mechanisms beyond social support is essential for improving mental well-being. Public health strategies should focus on providing comprehensive support, including unemployment benefits and access to mental health care, to mitigate the impact of unemployment on mental health.

## 5 Conclusion

This study examined the mediating role of perceived social support in the relationship between unemployment and mental distress among healthcare graduates in Bolivia during the COVID-19 pandemic. The findings revealed that while perceived social support generally improved mental health, it did not mediate the impact of unemployment on mental distress. Unemployment was identified as a significant risk factor for increased mental distress compared to employment. These findings highlight the importance of comprehensive public health strategies to mitigate the mental health impacts of unemployment among young healthcare graduates in LMICs, particularly during times of crisis.

## Data Availability

The datasets presented in this article are not readily available because of data protection reasons. Requests to access the datasets should be directed to the corresponding author.
