# Supplementary material for: Mediating role of perceived social support in the relationship between unemployment and mental distress among healthcare graduates during the COVID-19 era
Source: Front Public Health. 2025 Jan 28;12:1490004. doi: 10.3389/fpubh.2024.1490004 (PMC11810732; doi:10.3389/fpubh.2024.1490004)
Supplement: Supplementary file 1 [file Data_Sheet_1.pdf]

Supplementary Material

Supplementary Material

Table S1. Bivariate analysis by mental distress as binary variable (N = 109)

**Characteristics**	**Category**	**Missings (n = 8)**	**Mental Distress**		**p-values**
			**Yes (n = 69)**	**No (n = 32)**	
			**N (%)**		
Gender	Men	0	55 (79.7)	23 (71.9)	0.54
Career	Nursing	0	55 (79.7)	21 (65.6)	0.20
Economic situation	Good	3	12 (17.4)	1 (3.1)	0.11
	Neither good, nor bad		42 (60.9)	24 (75.0)	
	Bad		12 (17.4)	7 (21.9)	
Employment	Yes	3	35 (50.7)	10 (31.3)	0.11
			**Mean (SD)**		
Age		0	27.10 (2.30)	27.60 (4.10)	0.57

Chi-square test for gender, career and employment, fisher's exact test for economic situation, t-test for age

Table S2. Dimensions of precarious working conditions (Employment Precariousness Scale (EPRES)) of employed participants (N = 56)

**Subscale**	**Missings**	**Mean**	**SD**
Temporariness	3	1.41	1.39
Disempowerment	5	2.50	1.39
Vulnerability	4	1.47	0.93
Wages	3	1.81	0.55
Rights	3	1.47	1.10
Excercise rights	4	2.75	1.08
**Total**	**7**	**1.92**	**0.50**
		**Total**	**N (%)**
Level of precariousness[+]	7	(very) high	22 (44.9)
		Moderate to none	27 (53.1)

[+](very) high level of precariousness (LoP) with a total mean ≥ 2.00 and moderate to none LoP with a total mean < 2.00; SD = standard deviation

Table S3. Dimensions of perceived social support with MSPSS of all participants (N = 109)

**Items**		**Subscales**	**Missings**	**Mean (SD)**
1	There is a special person who is around when I am in need.	SO	18	4.58 (1.64)
2	There is a special person with whom I can share my joys and sorrows.	SO	18	4.73 (1.60)
3	My family really tries to help me.	Fam	18	5.00 (1.72)
4	I get the emotional help and support I need from my family.	Fam	18	4.97 (1.57)
5	I have a special person who is a real source of comfort to me.	SO	18	4.73 (1.71)
6	My friends really try to help me.	Fri	18	4.26 (1.51)
7	I can count on my friends when things go wrong.	Fri	19	4.27 (1.46)
8	I can talk about my problems with my family.	Fam	18	4.22 (1.77)
9	I have friends with whom I can share my joys and sorrows.	Fri	18	4.44 (1.50)
10	There is a special person in my life who cares about my feelings.	SO	19	4.62 (1.85)
11	My family is willing to help me make decisions.	Fam	18	4.75 (1.71)
12	I can talk about my problems with my friends.	Fri	18	4.22 (1.51)

MSPSS = Multidimensional Perceived Social Support Scale; SO = significant other, Fam = family, Fri = friends; Scores: 1 “very strongly disagree”, 2 “strongly disagree”, 3 “disagree”,

4 “neither agree nor disagree”, 5 “agree”, 6 “strongly agree”, 7 “very strongly agree”; SD = standard deviation. For subscales and total see Table 2.

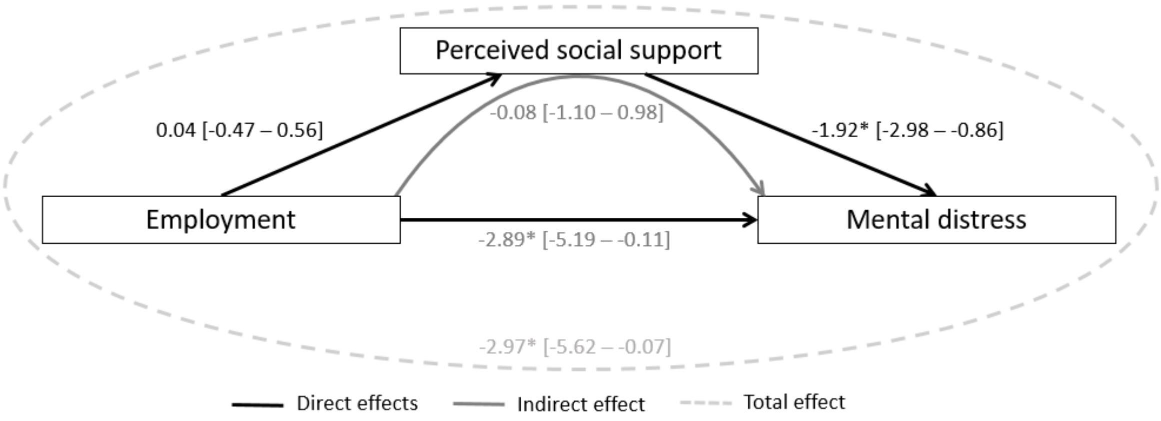

Controlled for gender and economic situation; *p-value < 0.05; mental distress: continuous variable; not imputed; 95% confidence interval in brackets; Direct effects: Measure the direct impact of employment on mental distress, excluding any mediation by perceived social support. Measure the direct impact of employment on perceived social support and the direct impact of perceived social support on mental distress; Indirect effect: Measures the impact of employment on mental distress through the mediator, perceived social support; Total effect: Captures the overall relationship between employment and mental distress, including both direct and indirect effects.

Figure S1. Sensitivity analysis with not imputed data: Mediating model of perceived social support between employment and mental distress (N = 89)

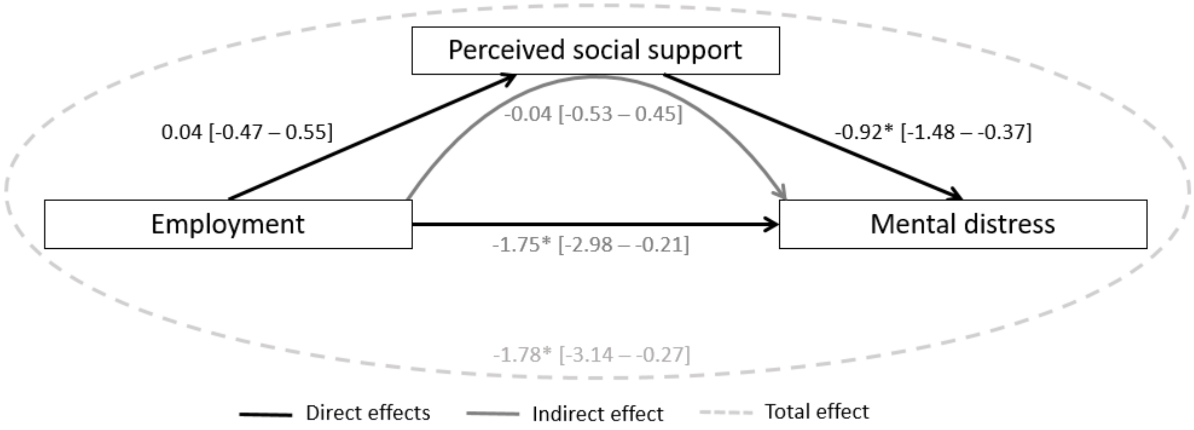

Controlled for gender and economic situation; *p-value < 0.05; mental distress: binary variable (threshold 4/5); imputed; 95% confidence interval in brackets; Direct effects: Measure the direct impact of employment on mental distress, excluding any mediation by perceived social support. Measure the direct impact of employment on perceived social support and the direct impact of perceived social support on mental distress; Indirect effect: Measures the impact of employment on mental distress through the mediator, perceived social support; Total effect: Captures the overall relationship between employment and mental distress, including both direct and indirect effects.

Figure S2. Sensitivity analysis with mental distress as binary variable: Mediating model of perceived social support between employment and mental distress (N = 109)

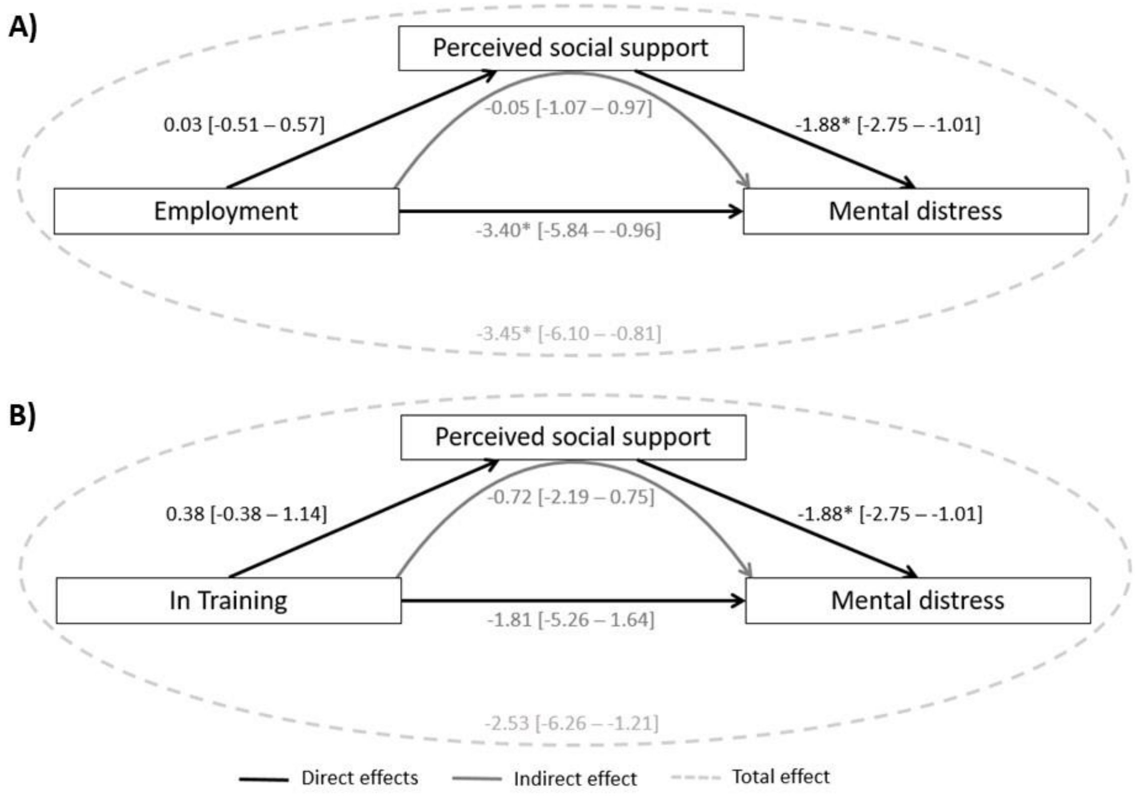

Controlled for gender and economic situation; *p-value < 0.05; mental distress: continuous variable; imputed; 95% confidence interval in brackets; A) participants, that are employed compared to those unemployed; B) participants, that are employed compared to those in training; Direct effects: Measure the direct impact of employment/training on mental distress, excluding any mediation by perceived social support. Measure the direct impact of employment/training on perceived social support and the direct impact of perceived social support on mental distress; Indirect effect: Measures the impact of employment/training on mental distress through the mediator, perceived social support; Total effect: Captures the overall relationship between employment and mental distress, including both direct and indirect effects.

Figure S3. Sensitivity analysis with training as additional category: Mediating model of perceived social support between employment/training and mental distress (N = 109)
